# Prevalence and associated factors of malnutrition among under-five children living in slum areas of Bahir Dar Town, Ethiopia

**DOI:** 10.11604/pamj.2024.47.176.33439

**Published:** 2024-04-09

**Authors:** Meron Tedla, Asmamaw Malede, Zemene Berhan

**Affiliations:** 1 Monitoring and Evaluation Office, West Gojjam Zone Health Department, Finote Selam, Ethiopia,; 2 Department of Environmental Health, College of Medicine and Health Sciences, Wollo University, Dessie, Ethiopia,; 3 Monitoring and Evaluation Office, Finote Selam General Hospital, Finote Selam, Ethiopia

**Keywords:** Prevalence, stunting, underweight, wasting, slum area

## Abstract

**Introduction:**

child malnutrition is one of the most serious and least addressed health problems in the world and in Ethiopia. The prevalence of malnutrition, underweight, and wasting was 44%, 29%, and 10% respectively. The Amhara region has the highest rates of malnutrition at 52%, 33.4%, and 9.9% for children under five. The aim of this study was to assess the prevalence of malnutrition and its associated factors among children under five living in the slum areas of Bahir Dar City.

**Methods:**

a community-based cross-sectional study was conducted with 680 children aged 6-59 months in slum areas of Bahir Dar Town. Study participants were selected using a mass sampling technique and data were collected from April to June 2018 using a pre-tested structured questionnaire and anthropometric measurements. Finally, the collected data were coded, entered, cleaned, recorded, and stored, and the data were processed using EPI INFO and exported to the SPSS version 25.0 statistical package. Logistic regression analysis and interpretation were performed using bivariate and multivariate analysis.

**Results:**

a total of 680 children participated. The prevalence of stunting, underweight, and wasting was 46.2% (95% CI; 42.5-49.1), 24.3% (95% CI; 21.2-27.6), and 11.3% (95% CI; CI; 9.2-13.9). Income groups included children [AOR=3.476 (95% CI, 1.959-6.167)], male children [AOR=2.586 (95% CI; 1.532-4.365)] and mother's educational level [(AOR=2.600) (1.623) - 4.164)] were significantly associated with malnutrition.

**Conclusion:**

the results of this study showed that the prevalence of malnutrition due to stunting and wasting was high among children under five years of age. The gender of the child, the educational level of the mother, and the monthly income of the family were found to be significantly related to malnutrition. Promoting the use of family planning, preventing diarrheal diseases, and vaccinating children through nutrition education programs are important activities to improve the nutritional status of children.

## Introduction

Malnutrition is one of the most important but neglected health problems in the world and the main cause of morbidity and mortality among children under 5 years of age in developing countries. It is associated with approximately half of all child deaths worldwide. Globally, approximately 60 million and 13 million children suffer from moderate and severe acute malnutrition, respectively, and 21.9%, 13.4%, and 7.3% of children under five are reported to be stunted, underweight, and wasted, respectively [1]. World Health Organization (WHO) estimates that approximately 5.4 million children under five die each year, and 2.7 million in sub-Saharan countries, including Ethiopia. Stunting and other forms of malnutrition reduce a child's chances of survival while preventing optimal health and growth. It has also been associated with suboptimal brain development, which is likely to have long-term negative consequences for cognitive abilities, school performance, and future earnings. This in turn affects the development power of the nation [2]. Malnourished children have reduced resistance to infections; they are more likely to die from common childhood diseases such as diarrhea and respiratory infections; and for those who survive, recurrent illnesses worsen their nutritional status, locking them into a vicious cycle of recurrent illnesses, stunted growth and reduced learning ability [3].

Children can become malnourished either because they do not eat enough food of the right form or quality, or because they are sick. The main causes of malnutrition at the household level are lack of food security, inadequate maternal and childcare practices, and poor health environment and services [4]. The impact of child care practices on children's well-being and the importance of child protection. Encouraging exclusive breastfeeding has gained increasing recognition in recent years. Breastfeeding is more nutritious, safer, and cheaper than bottle feeding, and it also increases babies' resistance to common childhood illnesses and infections. In addition, breastfeeding develops a healthy birth interval, which is a very important concern for the health of mothers and children. Although breastfeeding is affordable all over the world, it is especially important in the third world, where most families tend to be poorer, health services are lacking, and the general health environment is more dangerous [5].

In the case of rapid urbanization, 20% of city dwellers are forced to live in urban slums. Slums are the physical and social manifestations of the distribution of beneficiaries of economic growth and the structural activity and special model of the urban economy. The lack of human needs in urban slums negatively affects the growth and nutrition of slum dwellers. Children under the age of five living in slums are in a weaker position and are at greater risk of malnutrition due to low purchasing power, insufficient food consumption, and high infection burden [6]. In Ethiopia, nutritional status is the result of a complex interaction between food consumption, and health and healthcare practices. Many socio-economic and cultural factors influence the eating habits of children and the nutritional status of women and children.

According to the 2016 Ethiopia Demographic and Health Survey (EDHS) report, approximately 9.7% of children were wasted, 28.7% of children were underweight and 44.4% of children were stunted with large regional differences [7,8]. According to the Ethiopia Mini Demographic and Health Survey (EDHS) 2014 report, the drop rate among children under 5 is 40 percent. In the Amhara National Regional State of Ethiopia, stunting, wasting and underweight is 40%, 10%, and 33% respectively [9]. However, the nutritional status of children living in slum areas of Bahir Dar City was unknown. This study assessed the nutritional status of children under five living in the slum areas of Bahir Dar City.

## Methods

**Study area and time period:** the study was conducted in the slums of Bahir Dar City in April-June 2018. The city is the capital of the Amhara region and is located approximately 564 kilometers northwest of Addis Ababa (the capital), Ethiopia. Latitude and longitude of 11°36’N 37°23’W and a height of 1,840 meters above sea level, the city has a high temperature and an area of 213.43 square kilometers. According to a 2007 report by the Ethiopian Institute of Statistics, the city has a population of 221,991, of which 108,456 were males and 113,535 were females. The city is getting denser from time to time, which is why in different Kebeles you can observe big differences in the standard of living of people, which indicates the appearance of Kebeles slums. The city has nine suburbs, two of which are recognized as slums. In the slum areas of the city live 51,762 people, of which 23,366 were men and 28,396 were women. There are 3,988 children under the age of five. The city has 2 referral hospitals, 1 district hospital, and six health centers.

**Study design:** this was a community-based cross-sectional quantitative study design used to assess the nutritional status of children aged 6-59 months living in the slums of Bahir Dar City.

**Study population:** the target group of the study was all children aged 6-59 months, while the study population consisted of all households with randomly selected children aged 6-59 months living in Kebele. Participants who had lived in the study area for less than 6 months, seriously ill and difficult-to-communicate children and mothers, and children with physical deformities that prevented height measurement at the time of data collection were excluded.

**Sample size and sampling procedure:** sample size was determined using a single population proportion formula of Cochran, 1963 [10].


$$N=\frac{z^{2}\left(p\right)\left(q\right)}{{\left(e\right)}^{2}}$$

Where N = sample size, Z = standard error associated with the chosen level of confidence, p = variability/standard deviation (it can be taken from previous studies or pilot studies), q=1-p, and e= acceptable sample error. It is normally used at 0.05. Assume that, variability (p)= 0.5, confidence level (1-α)= 95%, and sampling error (e)= 5%, then:


$$N=\frac{{\left(1.96\right)}^{2}\left(0.5\right)\left(0.5\right)}{{\left(0.05\right)}^{2}}\approx 384$$

Considering the design effects of 1.5% and 10% non-response rates, the final sample size was 634 and was taken as the sample size for this study. A multistage cluster sampling method was used to recruit study participants in pastoralist communities. Two of the nine suburbs were slum areas included in our study. Then, the total number of children aged 6-59 months was taken from the households responsible for the selected kebeles through health center registration. The calculated sample size (634) was then distributed proportionally to the selected kebeles based on the total number of households with children aged 6-59 months in each kebele. Finally, after randomly identifying the first household, participants were selected using a systematic random sampling technique and transferred to the second participant based on the Kth time interval.

**Methods of data collection:** the questionnaire was developed based on the objectives of the study based on the Ethiopian Demographic and Health Survey (EDHS) [8] and other relevant literature. The questionnaire was translated into the local Amharic language for data collection. The study included socioeconomic and demographic factors, infant feeding and care practices, maternal health factors, environmental health characteristics, and anthropometric measurements. Data were collected from mothers of children aged 6-59 months using a structured questionnaire administered face-to-face by an interviewer. Ten registered nurses and four local language (Amharic) health officer supervisors, including the principal investigator, participated in the data collection process. Before the interview, informed consent was obtained from all participants after explaining the purpose of the study and confirming the confidentiality of the information.

All children aged 6-59 months were anthropometrically measured to assess their nutritional status. The height of a child aged 6-23 months was measured lying on a table with a vertical wooden base to the nearest 0.1 cm. The height of children aged 24-59 months was measured in an upright position to the nearest 0.1 cm using the same UNICEF-designed table as for the above age groups. The child's weight was measured in light clothing and without shoes to the nearest 0.1 kg using a UNICEF electronic scale. Children who could not stand during the measurement period were measured together with their mothers. The mother was weighed with and without the child, and then the mother's weight was subtracted from the total weight. The difference between the two measurements was the child's weight. The nutritional status of the children participating in the study was assessed using indicators according to age, weight, height, and height according to the WHO reference standard -2 S.D as the cut-off value indicating malnutrition (underweight, decrease, and weight loss).

**Data quality assurance:** the questionnaires were checked for completeness daily by supervisors and principal investigators on each day of data collection. After checking for consistency and completeness, the supervisors forwarded the completed questionnaires to the principal investigator. In addition, pre-testing of the study was conducted in 5% of the sample size in Kebele, where it was not part of the main study. The researcher in charge entered the collected data twice to check whether the data was entered correctly or not.

**Data management:** after data collection, each returned questionnaire was manually checked for completeness and consistency. The questionnaires were classified as incomplete, partially incomplete, and completed. Incomplete and partially completed questionnaires were excluded from the analysis. Only finished products were coded.

**Data processing and analysis:** data entry and analysis were performed using EPI data 3.1 and SPSS version 25.0. Anthropometric indices were calculated using the 2006 WHO Anthro 3.2.1 software. Descriptive analysis was used to describe the percentage and frequency of sociodemographic characteristics and other variables relevant to the study. Bivariate and multivariate logistic regression analysis was used to identify factors associated with child malnutrition. Both crude and adjusted probabilities and corresponding 95% confidence intervals were calculated to show the association between outcome and independent variables.

All independent variables associated with outcome variables (wasting, decrease and underweight) in bivariate analysis (p andlt; 0.25) were included in the final multivariate logistic analysis. P-value andlt; a result of 0.05 was considered statistically significant. The Hosmer-Lemeshow test was performed to examine model fit in the final model and p andgt; 0.05 was considered appropriate. The result was presented as text, tables, and graphs based on data types.

**Ethical considerations:** the study was approved by the Institutional Review Board of The Gambia College of Medicine and Health Sciences. Letters of authority have been obtained from the relevant authorities. Verbal consent was obtained from mothers or guardians. Privacy and confidentiality were maintained during the study period by omitting personal identifiers from the data collection forms. Malnourished children were referred to nearby health facilities for treatment.

## Results

**Socio-demographic characteristics:** a total of 680 children with their mothers from Kebele 2 in the sub-district of Gish Abay participated in the study. Three hundred and sixty-four of them (53.6%) were women. Most of them were Orthodox and Amharic by ethnicity. More than three-quarters of the mothers were married and cohabiting. About 74.9% of mothers lived in households with at least 4 family members and 86.6% of mothers had only one child under the age of five. About 80% of families live in a rented apartment. The majority of households, 440 (64.9%) received clean drinking water from water sources in the municipality. Almost 95% of mothers gave birth in health facilities. Five hundred and forty-two (79.7%) children were measured at birth according to the mother's data and records obtained from the cards and their return answers. About 11% of them were less than 2500 grams. The mean age of the children was 32.13 ± 15.08 months. There were 551 (81%) households with one child aged 6-59 months (Table 1).

**Table 1:** sociodemographic characteristics of children, in slum areas of Bahir Dar Town, June 2018

Variables	Category	Number (N=680)	Percent (%)
Sex	Male	316	46.5
	Female	364	53.5
Age of child	6-11 months	70	10.3
	12-23 months	148	21.8
	24-35 months	156	22.9
	36-47 months	166	24.4
	48-59 months	140	10.6
Vaccination status	Fully vaccinated	626	92
	Partially vaccinated	50	7.4
	Not vaccinated	4	0.6
Birth weight	Less than 2.5 kg	60	8.8
	Greater than 2.5 kg	481	70.8
	Un weighed	139	20.4
History (Hx) of diarrhea	Yes	60	9
	No	620	91
Hx of pneumonia	Yes	100	14.7
	No	580	85.3
Initiation of breast feed	Within 1 hr of delivery	573	84.3
	Above 1 hr of delivery	107	15.7
Initial time of complimentary feeding	Less than 6 months	33	4.9
	6 months-8 months	597	87.8
	Greater than 8 months	50	7.3

**Prevalence of malnutrition of under-five children:** the analysis of the three anthropometric indices stunting, wasting, and underweight showed that 46.2% (95% CI; 42.5-49.1), 24.2% (95% CI; 21.2-27.6) and 11.3% (95% CI; 9.2-13.9), were found to be stunted, underweight and wasted respectively. Among the total of 680 children included in the study, children aged 6 to 59 months, 371 (54.6%) were malnourished. There was difference between boys 151 (40.7%) and girls 220 (59.3%) (Table 2).

**Table 2:** sociodemographic characteristics of mothers, in slum areas of Bahir Dar Town, North West Ethiopia June 2018

Variables	Category	Number (N=680)	Percent (%)
Age of mother (years)	15-34	138	20.3
	25-34	372	54.7
	35-44	157	23
	45-54	13	2
Ethnicity	Amhara	662	97.4
	Agew Awi	9	1.3
	Tgrie	9	1.3
Religion	Orthodox	647	95.1
	Others	33	4.9
Education of mothers	Illiterate	291	42.8
	Grade 1-12	292	42.9
	College and above	97	14.3
Marital status	Married and lived in a union	545	80.1
	Single	135	19.9
Household monthly income	Lower (<1000 birr)	192	28.2
	Medium (1000-1800)	166	24.2
	Better (1800-2400)	161	23.7
	Higher (>2400)	161	23.7
House ownership	Private	145	21.3
	Rent	535	78.8
Water source	Private pipe	238	35
	Municipality pipe	442	65

**Stunting:** staggering slowed in 314 (46.2%) (95% CI; 42.5-49.1) of children aged 6-59 months. This proportion is lower in 25-year-olds (8%) and increases to about 12-23 months to 60-year-olds (19.5%) and is more common in the 24-35-month age group 86 (27.4%). There was a difference between the boys 215 (22.4%), and girls 102 (15%). Severe stunting (less than 3 SD) was present in 104 (15.3%) (12.8-18.2; 95% CI) of all children and shows a higher prevalence in boys 58 (18.2%) than women 48 (12.7%).

**Underweight:** of children aged 6-59 months, 165 (24.3%) were underweight. A total of 20 (2.9%) severely underweight followed similar patterns, such as deceleration, with boys having higher levels 14 (4.4%) than girls 6 (1.7%). As with stunting, younger children were less likely to be underweight than older children. The difference was between 88 (27.7%) boys and 77 (21.3%) girls. Children of highly educated mothers lost less 11 (14.3%) than children of uneducated mothers 10 (13%).

**Wasting:** seventy-seven (11.3%) of children aged 6-59 months were wasted. The difference was between 45 (14.2%) boys and 32 (8.8%) girls. A total of 35 (5.1%) had severe weight loss, and the difference between 26 (8.2%) boys and 9 (2.5%) girls is large. Similar to stunted and underweight, 7 (10.9%) younger children aged 6-11 months had less wasting than older children, for example, 21 years old (14.4%) in the age group 48-59 months. Children of highly educated mothers were less stunted 22 (13.3%) than children of illiterate mothers 34 (21%).

**Associated factors for stunting:** multivariate logistic regression analysis showed that family income, child's gender and mother's educational level were statistically related to stunting. Low-income children were 3.5 times more likely to be stunted than high-income children [adjusted odds ratio (AOR) = 3.476 (95% CI, 1.959-6.167)]. Similarly, children from middle-income families were twice as likely to have the disease as children from high-income families [AOR = 1.912 (95% CI, 1.143 to 3.200)], male children were approximately 3 times more likely to be stunted than females [AOR=3.043(95%CI, 2.141-4.326)]. Children of illiterate mothers were 3 times more likely to be stunted than children with higher education of mothers [AOR=2.930(95%CI, 1.730-4.964)] (Table 3).

**Table 3:** logistic regression analysis showing the association of selected socioeconomic and child health and care practice with stunting

Variables	Category	Frequency of stunting		COR (95%CI)	AOR (95%CI)	P-value
		Stunted	Normal			
Sex of child	Male	198	119	3.543(2.612-2.612)	3.043(2.141-4.326)	0.0001
	Female	116	247	1.00	1.00	
Family income	Lower (<1000)	86	60	4.483(2.863-7.020)	3.476(1.959-6.167)	0.0001
	Medium (1000-1800)	84	82	2.087(1.334-3.267)	1.912(1.143-3.200)	0.014
	Better (1800-2400)	45	116	0.791(0.491-1.272)	0.968(0.577- 1.626)	0.903
	Higher (>2400)	53	108	1.00	1.00	
Educational status of mother	Illiterate	183	108	3.002(1.861-4.840)	2.930(1.730-4.964)	0.0001
	Grade 1-12	96	196	0.868(0.536-1.404)	0.829(0.486-1.415)	0.492
	College and above	35	62	1.00	1.00	
Educational status of father	Illiterate	183	108	2.387(1.542-3.696)	0.877(0.476-1.614)	0.672
	Grade 1-12	136	140	1.308(0.933-1.835)	0.866(0.566-1.327)	0.509
	College and above	102	157	1.00	1.00	
Occupational status of father	Government	110	165	1.00	1.00	
	Private	126	153	1.261(0.900-1.766)	1.264(0.853-1.872)	0.243
	Daily laborer	71	44	2.421(1.569-3.735)	1.296(0.754-2.227)	0.348
Presence of ARI	Yes	58	42	1.747(0.220-0.457)	1.964(1.199-3.217)	0.007
	No	256	324	1.00	1.00	
House condition	Private	53	92	1.00	1.00	
	Rent	261	274	1.653(1.133-2.413)	1.000(0.632-1.584)	0.999

AOR: adjusted odd ratio; COR: crude odd ratio; ARI: acute respiratory infection

**Factors associated with underweight:** multivariate logistic regression analysis of underweight showed that family income and housing situation were statistically associated with underweight. Low-income children were 4.2 times more likely to be underweight compared to other income groups AOR=4.288(95%CI,2.352-7.817). Similarly, children whose family income was in the middle-income group were 2 times more likely to be underweight compared to the high-income group [AOR=2.072(95%CI, 1.113-3.858)]. Children of rental housing owners were 1.8 times more likely to be underweight than children of private homeowners [AOR = [95% CI, 1.841 (1.015-3.339)] (Table 4).

**Table 4:** logistic regression analysis showing the association of selected socioeconomic and child health and family factors with underweight

Variables	Category	Frequency of underweight		COR	AOR	P-value
		Underweight	Normal			
Family income	Lower (<1000)	85	107	5.937(3.401-10.363)	4.288(2.352-7.817)	0.0001
	Medium (1000-1800)	41	125	3.472(1.847-6.525)	2.072(1.113-3.858)	0.022
	Better (1800-2400)	20	141	1.060(0.543-2.071)	0.934(0.471-1.855)	0.846
	Higher (>2400)	19	142	1.00	1.00	
Occupation status of the father	Government	56	219	1.00	1.00	
	Private	59	221	1.054(0.699-1.589)	0.963(0.606-1.529)	0.872
	Daily laborer	49	66	2.619(1.649-4.160)	1.208(0.665-2.194)	0.534
Marital status of mother	Married and union	122	423	1.00	1.00	
	Single	43	92	1.621(1.071-2.452)	0.813(0.497-1.331)	0.411
Head of household	Male headed	116	411	1.00	1.00	
	Female-headed	51	104	1.768(1.192-2.622)	1.356(0.700-2.630)	0.367
House condition	Private	16	126	1.00	1.00	
	Rent	149	389	3.112(1.791-5.409)	1.841(1.015-3.339)	0.044
Family size	1-3	61	110	1.497(0.821-2.731)	0.918(0.478-1.764)	0.797
	4-6	84	351	0.646(0.367-1.138)	0.491(0.268-0.901)	0.22
	7-10	20	54	1.00	1.00	

AOR: adjusted odd ratio; COR: crude odd ratio

**Factors associated with waste:** multivariate logistic regression analysis showed that family income was statistically associated with waste. Children from low-income groups were 4.2 times more likely to lose than families with children from high-income families [AOR = 4.202 (95% CI, 1.879 to 9.393)] (Table 5).

**Table 5:** logistic regression analysis showing the association of selected socioeconomic, environmental factors child health and family factors with wasting

		Frequency of wasting		COR (95%CI)	AOR (95%CI)	P-value
Variables	Category	Wasted	Normal			
Family income	Lower	39	153	4.875(2.206-10.774)	4.202(1.879-9.393)	0.0001
	Medium	15	151	1.900(0.782- 4.613)	1.850(0.758-4.514)	0.176
	Better	15	146	1.965(0.809-4.773)	1.935(0.793-4.722)	0.147
	Higher	8	153	1.00	1.00	
Family size	1-3	34	137	2.375(1.001-5.638)	1.769(0.725-4.315)	0.210
	4-6	36	399	0.864(0.369-2.020)	0.770(0.324-1.828)	0.553
	7-10	7	67	1.00	1.00	
Occupation status of the father	Government	29	247	1.784(0.905-3.517)	1.263(0.585-2.726)	0.553
	Private	29	250	1.720(0.983-3.008)	1.123(0.547-2.306)	0.752
	Daily laborer	19	106	1.00	1.00	
Education status of the father	Illiterate	50	409	1.603(0.883-2.910)	0.911(0.423-1.959)	0.095
	Grade 1-12	22	122	1.720(0.983-3.008)	1.117(0.613- 2.037)	0.717
	College and above	5	72	1.00	1.00	
Head of household	Male headed	49	476	1.00	1.00	
	Female-headed	28	127	2.142(1.294-3.545)	1.396(0.800-2.438)	0.240
Marital status of Mother	Married and union	52	493	1.00	1.00	
	Single	25	110	2.155(1.281-3.624)	0.955(0.393-2.319)	0.919

NB: backward LR method was used to select predictors; AOR: adjusted odd ratio; COR: crude odd ratio

**Associated factors for malnutrition of under-five children:** multivariate logistic regression analysis showed that family income, child's gender, and mother's educational level were statistically related to malnutrition. Low-income children were 5.4 times more likely to be malnourished than high-income children [AOR=5.386 (95% CI, 2.981-9.730)]. Similarly, middle-income family incomes were 2.4 times more likely to be malnourished than high-income children. Higher-income groups [AOR = 2.481 (95% CI, 1.494-4.121)] males were approximately 2.6 times more likely to be malnourished than females [AOR = 2.566 (95% CI, 1.801-3.657)]. Children of illiterate mothers were 2.5 times more likely to be malnourished than children of mothers with higher education [AOR = 2.473 (95% CI, 1.468 to 4.165)] (Table 6).

**Table 6:** logistic regression analysis showing the association of selected socioeconomic, child health and care practice with malnutrition

Variables	Category	Frequency of malnourished		COR (95%CI)	AOR (95%CI)	P-value
		Malnourished	Normal			
Sex of child	Male	220	97	3.184(2.319-4.373)	2.566(1.801-3.657)	0.0001
	Female	151	212	1.00	1.00	
Family income	Lower (<1000)	153	39	6.782(4.214-10.915)	5.386(2.981-9.730)	0.0001
	Medium (1000-1800)	100	66	2.619(1.676-4.094)	2.481(1.494-4.121)	0.001
	Better (1800-2400)	59	102	1.935(0.793-4.722)	1.217(0.745-1.989)	0.432
	Higher (>2400)	59	102	1.00	1.00	
Education status of the mother	Illiterate	204	87	1.784(0.905-3.517)	2.473(1.468-4.165)	0.001
	Grade 1-12	121	171	1.720(0.983-3.008)	0.716(0.426-1.202)	0.206
	College and above	46	51	1.00	1.00	
Occupation status of the father	Government	134	142	1.00	1.00	
	Private	148	131	1.197(0.858-1.671)	1.177(0.799-1.734)	0.409
	Daily laborer	110	73	2.620(1.665-4.123)	1.195(0.680-2.098)	0.536
Education status of the father	Illiterate	86	39	2.554(1.628-4.007)	0.801(0.435-1.473)	0.475
	Grade 1-12	165	131	1.459(1.044-2.040)	0.945(0.621-1.440)	0.793
	College and above	120	139	1.00	1.00	
Family size	1-3	171	63	1.457(0.838-2.533)	0.781(0.409-1.490)	0.453
	4-6	435	212	0.894(0.545-1.466)	0.600(0.338-1.066)	0.082
	7-10	74	34	1.00	1.00	
Presence of Diarrhea	Yes	39	21	1.611(0.926-2.802)	1.127(0.595-2.137)	0.731
	No	332	288	1.00	1.00	
House condition	Private	58	84	1.00	1.00	
	Rent	313	225	1.897(1.308-2.751)	1.106(0.710-1.722)	0.655

AOR: adjusted odd ratio; COR: crude odd ratio

## Discussion

The overall results of this study showed that more than half of the children aged 6 to 59 months in the study were malnourished. The prevalence of malnutrition, stunting, underweight, and wasting in children aged 6-59 months was approximately 54.6%, 46.2%, 24.3%, and 11.3% respectively. Severe stunting, underweight, and wasting (less than -3 SD) were approximately 15.3%, 5.1%, and 2.9% respectively. These incidences of malnutrition, especially stunting and wasting, showed that the condition of children under five years of age in this study area was the worst compared to malnutrition reported in the Sidama zone, which showed 43 percent stunting and wasting of children (9.6%) [9]. In our study, the prevalence of stunting is higher than in a study done with a southern Ethiopian nationality [11]. This may be due to differences in the method used, differences in sample size, and low socio-economic and poor living standards in urban slum areas. The prevalence of stuttering in our study was approximately the same as in a study conducted in India, which is 48% (8) and lower than the result obtained in Nepal of 50.2 [12]. This may be due to socio-economic and cultural reasons differences between countries.

This research result shows that 24.3% of children aged 6-59 months were underweight. This finding was lower than the national prevalence (29%) and the regional prevalence of underweight (33.4%) [13]. This may be due to the small area of this study compared to the above regions and differences in sample size.

This finding indicates that 11.3% of children aged 6-59 months had vaping. This finding was higher than the regional (9.9%) and national figures (10%), indicating a serious problem in the study area during data collection [14]. This may be due to the living standards of the people in the area, cramped quarters, and poor hygiene practices that were practiced worse than elsewhere. The results of this study showed that the gender of the child, the educational level of the mother, and the monthly family income were significant related factors. Gender of the child. In this study, the prevalence of deceleration was higher in males than in females. Males were about 3 times more likely to be stunted than females. This finding was similar to the results of a study conducted in the Somali region, which showed that stunting is more common in boys than in girls [15]. Similarly, a study in Botswana showed that malnutrition is more common among boys than among girls [16]. This may be because men have a higher energy requirement than women. On the contrary, a study conducted in Nepal showed that malnutrition is higher among girls than boys [17].

Maternal education was significantly associated with stunting and was higher in children whose mothers were illiterate. Children of illiterate mothers were 3 times more likely to be stunted compared to children of mothers with a higher level of education. This may be because educated mothers have more knowledge about the importance of nutrition, personal hygiene, and preventive, supportive, and curative health services than uneducated or less educated mothers. So they pay attention to the food of their children. Similar findings have been reported in other studies. A study conducted in the Congo shows that children of educated mothers have less malnutrition than children of uneducated mothers [18]. Another study conducted in Bangladesh showed that children whose mothers had primary and secondary education had respectively 11 and 37 percent lower risk of birth defects than children of illiterate mothers [19]. Family/household income was significantly associated with malnutrition, underweight, and weight loss. The reason may be that the nutritional knowledge of people with higher incomes has improved and they are educated, and they have good opportunities to buy different foods for the household and have access to different foods at home. Family/household income was significantly associated with stunting. This is similar to a study done in Gondar [20].

Family income was significantly associated with underweight. A study conducted in the city of Al-Hilla showed that underweight children were significantly more common among children whose family income was insufficient [21]. A similar study in Kuwait showed that the prevalence of underweight was significantly related to family income. The highest family income level had low underweight compared to lower-income families. Family income was significantly related to waste. A study in Gumbrit showed that family income was significantly related to waste [22].

**Limitations of the study:** since the study involved a single cross-sectional design, the causal relationship between the dependent and independent variables may not be strong. It may also be possible to remember and report some indicators of infant and young child feeding (IYCF), such as breastfeeding habits, dietary diversity, and the child's past medical events.

## Conclusion

The results of this study showed that the prevalence of under-five malnutrition in stunted and wasted children was high. The gender of the child, the educational level of the mother, and monthly income of the family were found to be significantly related to malnutrition. Promoting the use of family planning, prevention of diarrheal diseases, and vaccination integrated into child nutrition education programs are important activities to improve the nutritional status of children. There were 51,762 communities living in slum areas; while eating, both male and female children should receive equal attention. Bahir Dar City health office; effective and long-term training in nutrition and nutrition practices should be provided, with an emphasis on infant and young child nutrition. Bahir Dar City Board of Education; emphasis should be placed on the empowerment of women by providing adult training for girls along with short-term specialized nutrition education to improve maternal nutrition practices.

### What is known about this topic

- *The results of this study showed that the prevalence of under-five malnutrition in stunted and wasted children was high, and the gender of the child, educational level of the mother, and monthly income of the family were found to be significantly related to malnutrition; promoting the use of family planning, prevention of diarrheal diseases, and vaccination integrated into child nutrition education programs are important activities to improve the nutritional status of children;*
- *Natural events such as droughts and floods, and conflict usually trigger food insecurity in Ethiopia, where over 85 percent of the population is dependent on rain-fed subsistence agriculture and livestock husbandry, resulting in an increased number of children with acute malnutrition; the study shows that there is high prevalence of malnutrition in Bahir Dar City if it is published it gives some clues for further investigation, and give real information for further study;*
- *Malnutrition in Bahir Dar City is high it revealed that in the slum areas, there is high stunting and underweight less than five children; it needs intervention*.

### What this study adds

- *Malnutrition is a big deal in a developing country such as Ethiopia, the burden is suffering every child everywhere, the evidence was not known, and the economic status, political, and social aspects were difficult for growth of the child, there were good clue for farther investigation;*
- *Regarding the slum area Bahr Dar is the capital city of the Amhara region which could be a model for preventing malnutrition but it could be suffering for such Burdon the research can give better information on what the government and the concerned body do;*
- *The research area was the regional city but there were many children suffering from malnutrition issues because of publication; the concerned body should be given attention to what they were not doing*.
